# Standard fractionation intensity modulated radiation therapy (IMRT) of primary and recurrent glioblastoma multiforme

**DOI:** 10.1186/1748-717X-2-26

**Published:** 2007-07-14

**Authors:** Clifton D Fuller, Mehee Choi, Britta Forthuber, Samuel J Wang, Nancy Rajagiriyil, Bill J Salter, Martin Fuss

**Affiliations:** 1 Department of Radiation Oncology, The University of Texas Health Science Center at San Antonio, San Antonio, TX, USA; 2 Graduate Division of Radiological Sciences, Department of Radiology, The University of Texas Health Science Center at San Antonio, San Antonio, TX, USA; 3 Department of Radiation Medicine, Oregon Health & Science University, Portland, OR, USA; 4 Department of Radio-Oncology, University of Innsbruck, Innsbruck, Austria; 5 Department of Internal Medicine, University of Texas Southwestern Medical School, Dallas, TX, USA; 6 Department of Radiation Oncology, University of Utah Health Sciences Center, Salt Lake City, UT, USA

## Abstract

**Background:**

Intensity-modulated radiation therapy (IMRT) affords unparalleled capacity to deliver conformal radiation doses to tumors in the central nervous system. However, to date, there are few reported outcomes from using IMRT, either alone or as a boost technique, for standard fractionation radiotherapy for glioblastoma multiforme (GBM).

**Methods:**

Forty-two patients were treated with IMRT alone (72%) or as a boost (28%) after 3-dimensional conformal radiation therapy (3D-CRT). Thirty-three patients with primary disease and 9 patients with recurrent tumors were included. Thirty-four patients (81%) had surgery, with gross tumor resection in 13 patients (36%); 22 patients (53%) received chemo-radiotherapy. The median total radiation dose for all patients was 60 Gy with a range from 30.6 to 74 Gy. Standard fractions of 1.8 Gy/day to 2.0 Gy/day were utilized.

**Results:**

Median survival was 8.7 months, with 37 patients (88%) deceased at last contact. Nonparametric analysis showed no survival difference in IMRT-boost vs. IMRT-only groups.

**Conclusion:**

While technically feasible, preliminary results suggest delivering standard radiation doses by IMRT did not improve survival outcomes in this series compared to historical controls. In light of this lack of a survival benefit and the costs associated with use of IMRT, future prospective trials are needed to evaluate non-survival endpoints such as quality of life and functional preservation. Short of such evidence, the use of IMRT for treatment of GBM needs to be carefully rationalized.

## Background

Malignant gliomas represent the most common primary brain tumors in adults, with approximately 75% of all gliomas classified as high-grade tumors. Within high-grade gliomas, Grade IV gliomas, or glioblastoma multiforme (GBM) exhibit a markedly more grim prognosis, with a median survival prognosis of 8 to 14 months [1-3].

The standard treatment of GBM includes surgical extirpation, followed by standard fractionation external beam radiation therapy [4]. When surgical resection is not feasible, radiation therapy is the primary treatment. Within the past decades several studies have explored new treatment regimens for GBM, mainly considering different combinations and doses of chemotherapeutic agents as well as various radiation therapy dose schema and delivery techniques [5-8].

Recently, novel radiation approaches affording increased dose-target conformality, such as intensity-modulated radiation therapy (IMRT), have been introduced. While IMRT may afford target isodose coverage superior to other external beam photon radiation techniques in scenarios involving geometrically complex target volumes adjacent to radiosensitive tissues, planning and delivery are resource intensive and require specific and costly software and hardware. As of today, the clinical feasibility and utility of IMRT techniques in GBM has not yet been fully elucidated. Also potential outcome benefits of this relatively novel delivery concept have not been assessed. This hypothesis generating retrospective study reports survival endpoint parameters of a consecutive series of patients with pathologically diagnosed GBM, treated with conventionally fractionated IMRT, delivered either as a monotherapy regimen or as a boost following conventional 3D-CRT.

## Methods

Chart review, data collection and analysis were approved by the Institutional Review Board of The University of Texas Health Science Center at San Antonio (IRB protocol # E-054-0242). Inclusion criteria for this retrospective chart review were pathological diagnosis of glioblastoma multiforme (WHO Grade IV) treated with IMRT using daily fractions between 1.8–2 Gy.

### Patient characteristics

Between January 1996 and January 2006, 42 patients with a pathological diagnosis of GBM completed a course of external beam radiotherapy (EBRT) either utilizing IMRT for the entire treatment course or as a boost following 3D-CRT. Of 42 patients, 30 (72%) received the entire treatment course by IMRT. Twelve patients (28%) were treated by IMRT delivered as a boost following three-dimensional conformal radiation therapy (3D-CRT). Thirty-three patients with primary disease and 9 patients with recurrent tumors were included in the study. All recurrent patients previously received radiotherapy, to a median dose of 52 Gy (range 36–62 Gy) at other institutions. For details on patient demographics please refer to Table 1.

**Table 1:** Patient demographic and treatment characteristics; percentiles are listed parenthetically.

**Characteristic**			**Series**	**Primary disease**	**Recurrent disease**
			(n = 42)	(n = 33)	(n = 9)
**Age (yrs)**					
	**Median**		60	63	46
	**Range**		20–86	40–86	20–59
					
**Sex**					
	**Male**		27 (64)	20 (60)	7 (78)
	**Female**		15 (36)	13 (40)	2 (22)
					
**Surgery**					
	**Biopsy alone/unresectable**		8 (19)	6 (19)	2 (22)
	**Debulking/resection**		34 (81)	27 (81)	7 (78)
		**Complete resection**	13(31)	10 (30)	3 (33)
		**Partial resection**	21(50)	17 (51)	4 (44)
					
**Chemotherapy**	**None**		19 (45)	17 (51)	2 (22)
	**Any agent**		23 (55)	16 (49)	7 (78)
		**Carmustine (iv)**	5 (12)	2 (6)	3 (33)
		**Carmustine (wafer)**	2 (5)	1 (3)	1 (11)
		**Lomustine**	1 (2)	1 (3)	-
		**Irinotecan**	3(7)	2 (6)	1 (11)
		**Penicillamine**	3 (7)	3 (9)	-
		**Temozolomide**	9(21)	7 (21)	2 (22)
					
**IMRT technique**					
	**IMRT Only**		30 (72)	22 (66)	8 (89)
	**3DCRT + IMRT boost**		12 (28)	11 (33)	1 (11)

### Radiation therapy simulation and target volume definition

Simulation was performed using a clinical CT simulator with helical image acquisition technique. For simulation, all patients were immobilized using a commercially available thermoplastic mask system (Raycast^©^-HP, Orfit Industries, Wijnegem, Belgium). Intra-venous contrast media was administered unless clinically contraindicated. CT image data were reconstructed in 2.5 or 3 mm slice thickness and co-registered with available MR image data in T2 or FLAIR (fluid attenuation inversion recovery) and T1 post-contrast weighting.

The initial clinical target volume (CTV) was defined as the hyper-intensity zone representing tumor and peri-tumoral edema plus margins of 2 cm on T2-weighted or FLAIR MR imaging. The target volume for the IMRT boost (CTV_boost_) included the contrast-enhancing region on T1-weighted MRI scans plus a margin of 10 mm. Also, organs at risk, such as the eyes, optic nerves, optic chiasm, and brainstem were delineated. CTV and CTV_boost _volumes were expanded into planning target volumes by adding 3-dimensional margins of 2 to 3 mm, values derived from an assessment of the immobilization accuracy of the aforementioned mask system [9].

### Treatment planning

The techniques employed for 3D-CRT varied slightly with each prescription. The predominant method of 3D-CRT delivery was a three-field technique (anterior-posterior and posterior-anterior field arrangement with a lateral oblique field) using 6 MV photons with custom blocking.

For inverse IMRT planning, the image datasets were transferred to the Corvus treatment planning system (Nomos Corp., Cranberry Township, PA). Inverse IMRT treatment planning requires the numerical entry of plan parameters into software templates. At a minimum, the target dose goal (prescribed dose, PD), the percentage of the target volume allowed to receive a lower dose (typically 5% or less), the minimum dose (95% of the PD) and the maximum dose (typically 107% of PD) desired for the target volume are entered. Similarly, a template for dose allowances and restrictions for organs at risk is populated. Dose prescription for the initial target volume was typically 45 to 46 Gy, with an additional dose of 14 or 14.4 Gy for the boost (total treatment dose of 59.4 or 60 Gy, in daily doses of 1.8 or 2 Gy).

The serial tomotherapy mode utilized for IMRT delivery in the present series treats the tumor in a rotational, slice-by-slice technique. Thus, the angle of rotation about the patients head (couch angle) and the range of rotation for each rotational arc were defined. In all cases presented here, the treatment was delivered as either a single arc, or with 2 couch angles, typically in a perpendicular arrangement (180 and 270 degree couch angle, Varian coordinates). The rotational arc was typically 340 degrees for the 180 degree couch and a shorter 210 degree arc for the 270 couch angle, to avoid collision with couch or patient.

The utilized binary multi-leaf collimator (MIMiC, Nomos Corp., Cranberry Township, PA) allows use of two generic pencil beam dimensions. All initial IMRT plans were computed using the so-called "2 cm" pencil mode (pencil beam dimension 17 × 10 mm); boost volumes were mostly treated using the smaller "1 cm" pencil beam mode (8.5 × 10 mm aperture). Treatment plans were optimized using a simulated annealing algorithm. All IMRT treatments were delivered using a 6 MV linear accelerator and the attached MIMiC binary multi-leaf collimator.

### Dose prescription

The median prescribed and delivered total dose for all patients was 60 Gy with a range from 30.6 to 74 Gy. Primary and recurrent tumors received a median total dose of 60 Gy, although the ranges differed slightly (56–74 Gy for primary disease, 30.6–74 Gy for recurrent tumors). The median total dose delivered by IMRT monotherapy was 60 Gy (range 30.6–72 Gy). The median total dose in patients receiving IMRT as a boost was 66.6 Gy (range 56–74 Gy). Standard fractions of 1.8 Gy to 2.0 Gy/day were utilized.

### Follow-up

Follow-up evaluations were performed 6 weeks after completion of therapy and every 3 months thereafter. No patient was lost to follow up.

### Data analysis

Collected data regarding clinical, treatment, and survival parameters were analyzed using JMP statistical software (SAS Institute, Cary, NC). Kaplan-Meier survival analysis was performed using survival data. Non-parametric statistical techniques were utilized for comparative analysis, as dictated by group size and non-Gaussian distributions.

## Results

**Figure 1:**
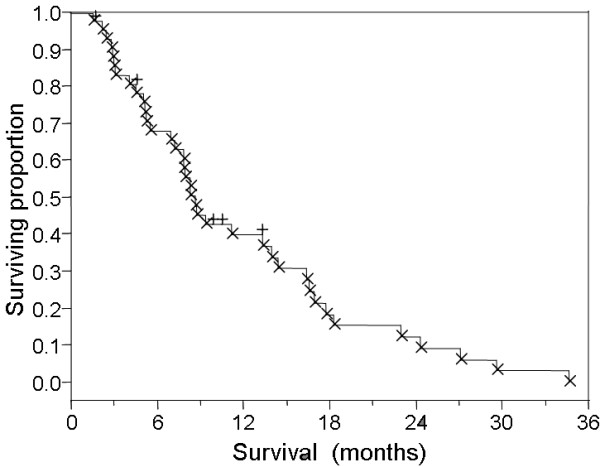
Kaplan-Meier overall survival for all 42 patients (+ = alive at last contact, x = deceased).

**Figure 2:**
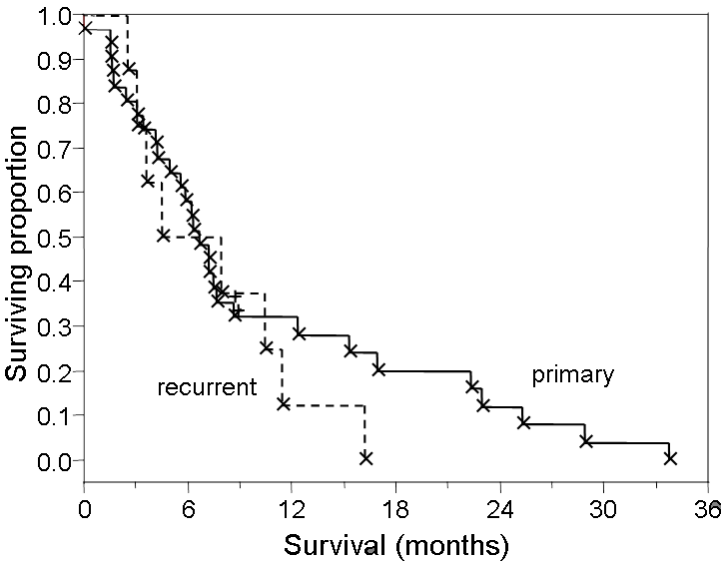
Survival from inception of radiotherapy for primary (solid line) and recurrent (dashed line) disease.

**Figure 3:**
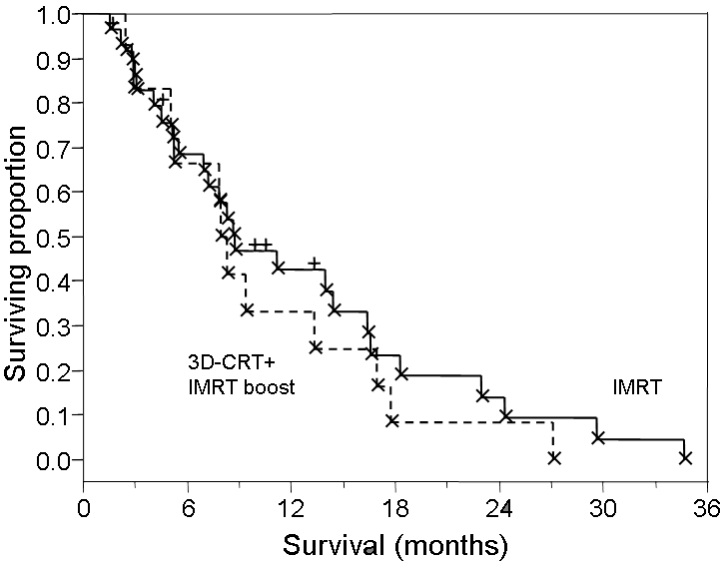
Survival for patients treated with IMRT alone (solid line) or 3D-CRT with IMRT boost (dashed line).

Of 42 patients, 37 (88%) were deceased at last contact, with a median survival of 8.7 months (range 1.6–34.7 months, Figure 1). Median survival from the initiation of radiation therapy for recurrent GBM was 4.5 months (range 1–16.2, Figure 2). No survival differential was detected between cohorts receiving 3D-CRT with IMRT-boost or IMRT as monotherapy (logrank and Wilcoxon analysis). Wilcoxon and Kruskal-Wallis nonparametric analysis of correlation between total dose delivered, and proportion of therapy delivered with IMRT revealed no detectable difference in survival between IMRT-boost and IMRT-only groups (Figure 3). Progression-free survival was calculated as time from diagnosis to either radiologic progression or demise. A median progression free survival period of 7.2 months (range 1.0–34.7) was observed for the entire series, with median time to progression from diagnosis of 7.3 months for patients with primary disease compared to a median PFS of 4.5 months in recurrent GBM patients (p = 0.2, non-significant).

Maximum acute treatment-attributable toxicity, by RTOG Acute Toxicity Score is listed in Table 2. Five patients (12%) exhibited greater than RTOG Grade 2 treatment-related toxicity, specifically acute hemiplegia in 3 patients requiring hospitalization, and seizure requiring hospital admission in 2 patients. In no case was therapy aborted prematurely secondary to acute toxicity attributable to therapy.

**Table 2:** RTOG Maximum acute toxicity score by cohort; percentiles are listed parenthetically.

Maximum RTOG Acute Toxicty	Series	Primary disease	Recurrent disease	IMRT	3DCRT+IMRT boost
0	6(14)	6 (18)	0 (0)	5 (16)	1 (8)
1	11 (26)	9 (27)	2 (22)	6 (20)	5 (42)
2	20 (48)	14 (42)	6 (67)	15 (50)	5 (42)
3	5 (12)	4 (12)	1 (11)	4 (13)	1 (8)

## Discussion

While multimodality therapy has been demonstrated to improve overall survival of patients diagnosed with glioblastoma multiforme compared to surgery alone[4], there is no established schema that has proven optimal for treatment of GBM. GBM is notoriously refractory to therapy, with survival rarely exceeding 2 years. More than 95% of patients with primary GBM receiving an initial therapy of surgery and external beam radiotherapy(EBRT) with or without concomitant and/or adjuvant chemotherapy, fail within 5 years, and recent literature suggests that even this slim margin of survival may be exaggerated[10].

Several studies have concluded that local tumor progression was the predominant pattern of failure [11-13]. The observation that the vast majority of recurrences are focal, at the initial site of the neoplasm[14], has provided an impetus for dose delivery to reduced radiotherapy volumes. Subsequent technological advances in external beam radiation therapy have resulted in investigation into more tumor-conformal radiation delivery techniques, such as 3D-CRT. These techniques spare normal brain tissue from the high-dose area of radiation and can theoretically afford higher radiation dose delivery to brain tumors safely. In an effort to explore dose escalation with 3D-CRT, Nakagawa et al. studied survival in GBM patients using multi-leaf collimator conformal radiation therapy[1]. Approximately 55% of the patients were treated to a dose of 60 to 80 Gy and 44% were treated to 90 Gy in addition to intravenous chemotherapy, which resulted in an alteration of patterns of failure, but no discernable survival benefit.

The inception of IMRT brought with it great optimism with regard to brain tumors, as the radiation dose conformality available with IMRT is unparalleled[15,16]. However, since the development of IMRT in the 1990s, few studies in the literature have assessed the survival impact of this radiotherapy modality with regard to GBM. In a recent series, Narayana et al. report on 41 glioblastoma cases out of a total of 58 high grade gliomas treated with standard fractionation IMRT at Memorial Sloan-Kettering Cancer Center[17]. This series exhibited exceedingly similar results to the current series, with reported overall survival of 9 months for glioblastoma. While the Memorial group used dynamic leaf IMRT, in contrast to serial tomotherapeutic IMRT utilized in the present series, we believe that the nearly equivalent results from both series, in similar numbers of patients, treated with similar dose parameters, add confirmation to the findings reported here.

The majority of other reports of GBM treated with IMRT involve altered fractionation schedules, with the intent of using the amenity of IMRT dose shaping to minimize adjacent tissue dose while maximizing radiobiological parameters in an effort to improve tumor dose in primary tumors. However, the results from such series have failed expectations with regard to added survival. Thilmann et al[18] examined the feasibility and safety of an integrated IMRT boost in addition to conventional EBRT in 20 patients, and, though survival data is not yet available, a Phase III trial is underway. Suzuki et al [19] also studied the feasibility of an integrated boost method using intensity modulated radiotherapy (IMRT). The total dose delivered was 70 Gy in 28 fractions of 2.5 Gy. No delay in therapy from radiation toxicity was necessitated in any of the 6 enrolled patients. Sultanem et al[20] recently published data from a series of 25 patients treated with hypofractionated IMRT (60 Gy in 3 Gy increments). Median survival in said study was 9.5 months, consonant with the survival observed in the present study.

In addition to individuals with primary disease, the conformal dosimetric profiles attainable with IMRT have been examined as a means of treating recurrent GBM. Voynov et al[21] record a series of 10 patients for whom stereotactic IMRT using serial tomotherapy was implemented in an effort to treat recurrent malignant gliomas, resulting in a median overall survival time of 10 months, and 50% and 33.3% one and two year survival, respectively. The data derived from the present series reveals slightly inferior outcomes for recurrent disease, with median survival of <5 months.

To our knowledge, this dataset represents one of the few extant series of GBM patients treated with standard fractionation IMRT alone, as well as the largest retrospective study to date of survival data with a 3D-CRT/IMRT boost technique. Our study revealed no substantial differential in survival times of patients treated with IMRT conformal techniques from reported survival in the literature of patients treated with conventional methods, and is similar to recent studies exploring alternative fractionation IMRT methodologies [18-20]. There are several possible explanations for this observation. Admittedly, this review is retrospective and numerically limited, with several heterogenous treatment schemas. Additionally, dose escalation was not a primary focus of treatment regimens within this series, nor was fraction-size optimization. These caveats draw attention to the necessity of standardized trials designed to optimize the dosage and fractionation schedules utilized in the treatment of GBM. Additionally, while survival was the primary end-point of note in this study, definitive explication of the role of conformal techniques in non-mortality endpoints, such as disease progression, metabolic or anatomical imaging parameters, neurocognitive outcomes, toxicity reduction and/or quality of life enhancement must be explored as they may represent a more realistic aim in IMRT-based interventions than extension of mortality[22,23].

Nonetheless, the preliminary results of our analysis suggest that undue optimism regarding reduction in mortality for GBM treated with IMRT therapies must be tempered by the recalcitrance of this tumor to radiotherapy. No extant IMRT series to date has matched the survival outcomes observed in the control arm of the EORTC trial[24], where a 12.1 month median survival using 60 Gy in 2 Gy fractions delivered using quality-assured 3D-CRT was achieved. Consequently, it remains to be seen whether the addition of IMRT will improve upon the impressive 14.6 month median survival seen in the same study's experimental arm of 3D-CRT plus concurrent temozolomide.

## Conclusion

Conscientious practice requires justification for a technique which is markedly more complicated and economically costly, and has not, at present, demonstrated conferral of a statistical survival greatly distinct from conventional series. If, as our data suggests, standard fractionation IMRT does not provide an imminent and effective benefit in terms of mortality, careful thought must be made to widespread initiation of treatment protocols whose dollar costs are several times that of conventional techniques, with questionable therapeutic advantages[17]. It is our belief that elucidation of the potential role of IMRT and other conformal therapies is best accomplished through standardized clinical trials and multi-institutional cooperative group studies, exploration of multi-modality chemoradiation or alternative fractionation protocols.

## Abbreviations

glioblastoma multiforme (GBM)

intensity modulated radiation therapy (IMRT)

three-dimensional conformal radiation therapy (3D-CRT)

external beam radiotherapy (EBRT)

magnetic resonance imaging (MRI)

## Competing interests

BS, M.F, Nomos/North American Scientific, Chatsworth, CA, F. Consultant/Advisory Board.

## Authors' contributions

CDF and BF conceived of the study, collected data, performed statistical analysis, and drafted the manuscript. MC and SW collected data, participated in coordination and statistical analysis, and helped to draft the manuscript. NR and BS and participated in the design of the study and assisted in data collection statistical analysis. MF oversaw project completion, provided mentorship, participated study design and coordination, edited the manuscript. All authors have read and approved the final manuscript.
